# Translating Biomarker Research into Clinical Practice in Orthopaedic Trauma: A Systematic Review

**DOI:** 10.3390/jcm14041329

**Published:** 2025-02-17

**Authors:** Alexander Baur, Augustine Mark Saiz

**Affiliations:** 1 Liberty University College of Osteopathic Medicine, Lynchburg, VA 24502, USA; 2 Department of Orthopaedic Surgery, UC Davis Health, Sacramento, CA 95817, USA

**Keywords:** orthopaedic trauma, damage control orthopaedics, early appropriate care, biomarkers, surgical timing

## Abstract

**Background/Objectives:** Orthopaedic trauma management in polytrauma patients presents challenges, particularly in selecting between damage control orthopaedics (DCO) and early appropriate care (EAC). This systematic review evaluates these approaches and explores the role of biomarkers in optimising surgical timing. The primary objective of this review was to evaluate the potential clinical utility of biomarkers in guiding surgical timing and predicting perioperative complications. The secondary objective was to compare the effectiveness of DCO and EAC approaches, focusing on their impact on patient outcomes when controlled for Injury Severity Scores (ISSs). **Methods:** A systematic search of PubMed, MEDLINE, and Google Scholar identified studies focusing on fracture management (DCO versus EAC), timing protocols, and biomarkers in polytrauma patients. Twenty-seven studies met inclusion criteria. **Results:** Among the 27 studies, 12 evaluated biomarkers and 15 compared DCO and EAC. Point-of-care (POC) biomarkers, including lactate (*p* < 0.001; OR 1.305), monocyte L-selectin (*p* = 0.001; OR 1.5), and neutrophil L-selectin (*p* = 0.005; OR 1.56), demonstrated predictive value for sepsis, infection, and morbidity. CD16bright/CD62Ldim neutrophils were significant predictors of infection (*p* = 0.002). Advanced biomarkers, such as IL-6, IL-10, RNA IL-7R, HMGB1, and leptin offered prognostic insights but required longer processing times. No clear superiority was identified between DCO and EAC, with comparable outcomes when injury severity scores (ISS) were controlled. **Conclusions:** This systematic review highlights the challenge of translating biomarker research into clinical practice, identifying several point-of-care and advanced laboratory biomarkers with significant potential to predict complications like sepsis, infection, and MODS. Future efforts should focus on refining biomarker thresholds, advancing point-of-care technologies, and validating their role in improving surgical timing and trauma care outcomes.

## 1. Introduction

Orthopaedic trauma management in polytrauma patients presents a multifaceted challenge, where timely and precise interventions are paramount for achieving optimal outcomes. The decision-making process surrounding fracture management in these patients is particularly complex, requiring a delicate balance between immediate stabilisation and the acute consequences of surgical intervention [1].

The global burden of orthopaedic and musculoskeletal trauma is profound, affecting millions annually and representing a significant source of morbidity and socioeconomic strain [2]. Trauma-related injuries account for substantial disability-adjusted life years, with road traffic accidents and falls being among the leading causes. Miclau et al. emphasize that while advancements in trauma care systems have improved outcomes in high-income countries, the disparities in low- and middle-income regions remain stark, where limited access to trauma systems, prehospital care, and trained surgeons exacerbates the burden [3]. Even in high-income countries, Hoogervorst et al. highlight the substantial economic toll of musculoskeletal injuries, as both direct healthcare costs and productivity losses significantly impact individuals and national economies [4]. As Stinner and Edwards discuss, there is a substantial need for coordinated and comprehensive trauma systems [5]. Implementing well-designed systems that integrate the most up-to-date research can enhance acute care outcomes while reducing both mortality and long-term disability associated with these injuries. Globally, enhancing trauma care accessibility and standardisation holds the potential to significantly mitigate the impact of musculoskeletal trauma on patients and society.

Trauma resuscitation has been a cornerstone of critical care research for decades, with a primary focus on stabilising patients and mitigating life-threatening physiological derangements [6]. Historically, metrics such as lactate clearance and base deficit have served as reliable indicators of resuscitation efficacy, correlating with outcomes such as organ dysfunction and mortality [7,8]. These foundational studies have established the importance of achieving optimal physiological stability before proceeding with surgical interventions.

The evolution of trauma care has shifted from focusing solely on achieving resuscitation endpoints that are concrete to a more comprehensive and fluid understanding of the physiological response to trauma. Recent advancements in medical science, particularly in understanding the physiologic response to trauma, offer promising avenues to refine the decision-making process in orthopaedic trauma management [9,10]. Biomarkers such as interleukin-6 (IL-6) and neutrophil L-selectin show potential in predicting postoperative complications and guiding optimal timing for surgical interventions. By integrating biomarker data into clinical practice guidelines, clinicians may enhance the precision and safety of surgical timing, thereby potentially reducing risks associated with either premature or delayed procedures [11]. Lord et al. and Weinberg et al. underscore the growing body of evidence supporting the integration of resuscitation metrics within the broader physiological context, highlighting this as a critical area of research in trauma care [12,13]. Their comprehensive systematic reviews emphasise the importance of this shift in guiding informed and adaptive decision making, particularly in determining the optimal timing for surgical interventions. This evolving focus not only underscores the relevance of this research but also lays the foundation for advancing clinical practices, supporting the central themes of this paper.

While acid–base measurements remain the most researched and primary indicators, they are now understood to function within a more fluid and dynamic context that includes systemic inflammation, immune dysregulation, and metabolic derangements. This broader understanding has significant implications for trauma management strategies, particularly in orthopaedics, where decisions about surgical timing and the approach to injury fixation—whether through temporary stabilisation or definitive repair—are critical.

Two primary strategies have emerged: damage control orthopaedics (DCO) and early appropriate care (EAC). DCO involves initial, temporary acute stabilisation without definitive reduction and fixation in order to mitigate systemic inflammatory responses and physiological stress, deferring definitive surgery until the patient’s condition stabilises [9,14]. Conversely, EAC aims for prompt anatomical alignment and functional restoration through early comprehensive surgical repair. The controversy between DCO and EAC revolves around their respective benefits and risks, with extensive research exploring which approach yields superior outcomes under varying clinical scenarios. While each method has merits, the choice hinges on patient-specific factors, injury severity, and the patient’s overall physiological status. Central to this decision-making process is the concept of physiological optimisation, as the patient’s overall stability and response to resuscitation ultimately dictate whether to proceed with DCO for temporary stabilisation or EAC for definitive surgical repair.

The primary objective of this systematic review is to evaluate current practices in orthopaedic trauma management, focusing on the role of biomarkers in optimising surgical timing and outcomes. The secondary objective is to compare the effectiveness of DCO versus EAC in relation to patient physiology, injury severity, and clinical protocols.

## 2. Methods

This systematic review adhered to the Preferred Reporting Items for Systematic reviews and Meta-Analyses (PRISMA) 2020 guidelines (See supplementary checklist in Supplementary Materials) [15]. The review was registered with PROSPERO (CRD42024528619) before commencement to ensure originality and adherence to systematic review standards.

Our research questions were: (1) Can point-of-care biomarkers help predict perioperative complications in polytrauma patients undergoing orthopaedic surgery? (2) How do advanced immune biomarkers compare in their ability to predict surgical outcomes and guide timing decisions? (3) What is the comparative effectiveness of damage control orthopaedics (DCO) versus early appropriate care (EAC) when accounting for injury severity? These questions aim to clarify the role of biomarkers in clinical decision making and evaluate surgical strategies to improve patient outcomes.

The inclusion criteria for the review were determined by a two-stage screening process, where two reviewers independently screened each record and report retrieved; any discrepancies were resolved by the senior author, ensuring consensus. Inclusion criteria required studies on orthopaedic trauma in adult polytrauma patients, specifically evaluating fracture management or orthopaedic interventions and reporting on timing protocols or biomarkers. Eligible study designs included RCTs, cohort studies, and case–control studies in peer-reviewed English-language journals.

Exclusion criteria included studies on non-traumatic orthopaedic conditions, non-orthopaedic trauma, isolated spinal fractures, proximal femur fractures, or those lacking direct relevance to fracture management. Studies focused solely on imaging were also excluded.

**Figure 1:**
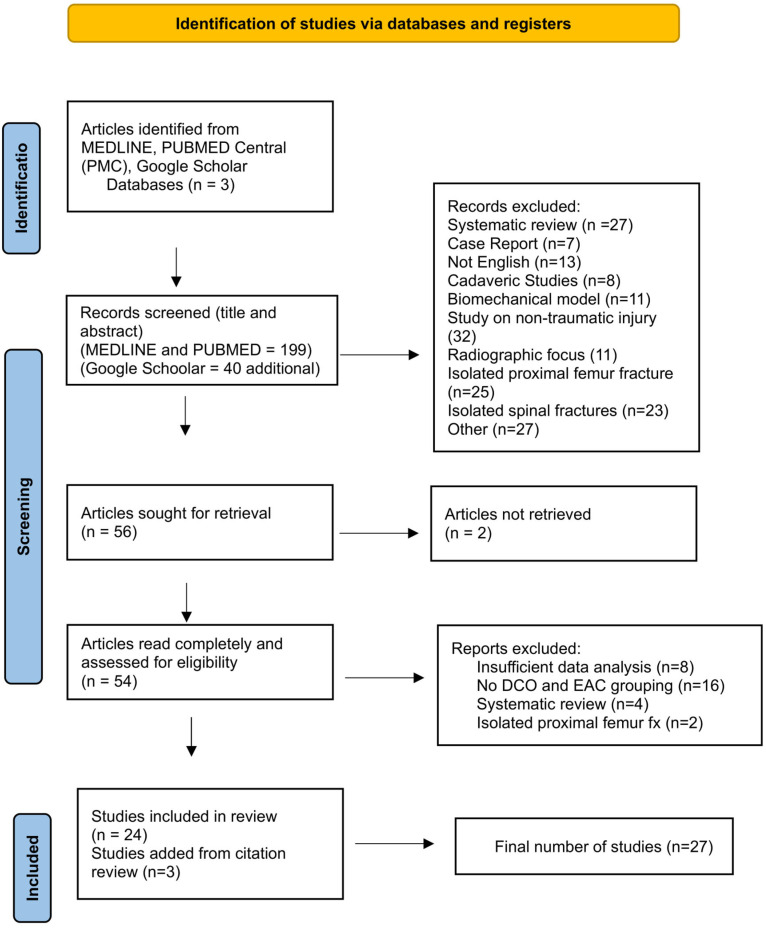
PRISMA flow diagram.

A comprehensive search was conducted using electronic databases, including PubMed, MEDLINE, and Google Scholar. Specific search strategies for each database can be found in Supplemental File S1. For each search, the following search terms were used (Dec 2024): ((orthopedic trauma) OR (orthopedic injuries)) AND ((fracture management) OR (orthopedic surgical procedures) OR (orthopedic interventions)) AND ((optimal timing) OR (biomarkers) OR (Damage Control) OR (Early Appropriate Care)) AND ((clinical outcomes) OR (fracture nonunion) OR (complications)) AND (polytrauma) NOT (spine or imaging). The PRISMA 2020 flow diagram (Figure 1) for the systematic review details the processes used to identify and assess eligible studies. Titles were independently screened by two authors, resulting in 239 articles eligible for abstract screening across the three databases. Abstracts were then reviewed in full against the inclusion and exclusion criteria (see Figure 1 for exclusions in this stage of screening). After this screening process, 56 articles were selected for full-text retrieval. Of the 56 articles sought for retrieval, two were not retrievable, leaving 54 articles to be fully reviewed and assessed for eligibility. At this stage, reports were excluded because they focused on proximal femur fractures (2), lacked sufficient data analysis (8), did not include DCO and EAC grouping (16), or were systematic reviews (4). Additionally, reference lists of eligible articles were analysed, resulting in the inclusion of three additional studies, culminating in a final total of 27 studies for synthesis.

After selecting 27 papers for inclusion, both authors independently assessed the risk of bias for each study using the ROBINS-I tool (Table 1 for non-randomised trials and the RoB version 2 tool (Table 2) for randomised trials [16,17]. Any discrepancies were resolved through discussion, and no automation tools were used in this process.

We systematically extracted data on clinical, functional, intraoperative, postoperative, and additional variables to ensure a comprehensive analysis. Data from each report were collected independently by two reviewers and recorded in Microsoft (One Microsoft Way, Redmond Washington 98052 USA) Excel 365 Version 2024, with any discrepancies resolved through discussion. Clinical outcomes included infection rates, delayed union, and nonunion. Intraoperative data encompassed OR time, blood loss, and transfusion requirements. Postoperative outcomes included ICU and hospital LOS, ventilation hours, and transfusions. Functional recovery was assessed using patient-reported outcomes, while mortality was recorded at in-hospital, 28-day, and long-term intervals. Complications included DVT, sepsis, MOF, ARDS, and cardiac events. Any missing or unclear information was addressed by cross-referencing related sections of the reports or consulting with senior investigators.

Results from individual studies were tabulated to display intervention characteristics and outcomes systematically. The summary table was further divided after data extraction to provide clear comparisons between studies. These sub-groups added to the overall analysis and study heterogeneity.

## 3. Results

Our systematic review included 27 studies in total, 12 of which focused on biomarkers illustrated in (Table 3). The primary objective of this systematic review was to identify clinically relevant biomarkers that can guide surgical treatment and decision making in trauma patients. The aim was to determine which biomarkers have the greatest potential to inform timing and strategies for surgical interventions in this population.

Our systematic review identified two broad categories of biomarkers: POC and advanced laboratory studies. The POC biomarkers provide rapid results, typically within 30–45 min, whereas the advanced laboratory studies such as ELISA, PCR, or flow cytometry may take much longer. When looking at the POC tests, Briggs et al. identified monocyte L-selectin as predictive of sepsis (*p* = 0.001, odds ratio [OR] = 1.5), along with neutrophil L-selectin (*p* = 0.005, OR = 1.56) [40]. Spijkerman et al. reported that CD16bright/CD62Ldim neutrophils were predictive of infection (*p* = 0.002) [11]. The most prominent POC biomarker our study identified was lactate, which was evaluated in several studies. Oladipo et al. showed that lactate was associated with post-operative morbidity (*p* = 0.015, OR = 1.305) and length of stay (LOS) (*p* < 0.001) [45]. Nishida et al. demonstrated that lactate was significantly associated with post-operative complications (*p* = 0.04, OR = 2.64), although there was no significant association with LOS (*p* = 0.78) [46].

Advanced laboratory biomarkers required specialised testing methods such as ELISA, PCR, or flow cytometry, with results taking 4–6 h to process. Gaski et al. found that IL-10 (*p* = 0.01), HMGB1 (*p* = 0.03), and MIG (*p* = 0.05) were significantly predictive of nosocomial infections [24]. IL-6, IL-8 IL-1RA, and MCP-1 were all significantly associated with the degree of organ dysfunction (*p* = 0.0001, *p* = 0.015, *p* = 0.13, and *p* = 0.08, respectively) [24]. Haupt et al. reported that leptin (*p* = 0.027) and IL-17A (*p* = 0.02) were associated with MODS [35]. Jin et al. showed that RNA IL-7R was a highly accurate predictor of MODS (*p* = 0.001) [36]. Frohlich et al. demonstrated that CD144+ and CD42B+ were significantly correlated with injury severity scores (both *p* < 0.001) [34].

The secondary objective of this study was to evaluate if DCO or EAC lead to superior outcomes in orthopaedic trauma patients, aiming to determine if the choice of strategy significantly impacts patient outcomes. Among the 15 studies comparing DCO and EAC (Table 4), 3 were randomised controlled trials, 10 were retrospective cohort studies, 5 were cohort studies (4 retrospective and 1 prospective), and 7 were case–control studies. Collectively, these studies provided comprehensive insights into the timing of orthopaedic trauma management, encompassing a total patient population of 35,026. The findings aimed to assess whether different approaches to surgical timing influence key outcomes such as complication rates, recovery trajectories, and overall patient prognosis.

Across the studies reviewed, Injury Severity Scores (ISSs) were consistently higher in patients treated with DCO compared to EAC, even in studies employing matched cohorts or attempting to balance patient groups. For instance, Andruszkow et al. reported ISS values of 34.4 in the DCO group versus 25.5 in the EAC group (*p* < 0.001) in their German cohort and 41.0 versus 34.0 (*p* < 0.001) in their Australian cohort [32]. Similarly, Lubken et al., in a large cohort of over 12,000 patients, observed mean ISS values of 30.5 for DCO versus 25.9 for EAC (*p* < 0.001) [21]. These findings reflect the consistent use of DCO in patients with higher trauma severity, even when attempts were made to control for injury severity.

Interestingly, studies with lower overall ISS values in both groups still demonstrated significant differences between DCO and EAC. For example, Li et al. reported mean ISS values of 28.1 for DCO and 21.3 for EAC (*p* < 0.001) [18]. These results highlight that even in less severely injured populations, there remains a clear delineation in the severity of injuries between patients treated with DCO versus EAC, which may be institution dependent.

Of the studies reviewed, only four reported no significant difference in Injury Severity Scores (ISS) between DCO and EAC groups: two randomised controlled trials (Pape et al., 2003; Rixen et al., 2016), one retrospective cohort study (Yu et al., 2023), and one retrospective comparative study (Enninghorst et al.) [25,30,37,39]. These studies are particularly notable because they eliminate the confounding effect of differing injury severity, making their findings more relevant for guiding decision making in borderline cases where patient stability might allow for either approach.

In these studies, outcomes between DCO and EAC were generally comparable, as demonstrated by Table 5. Pape et al. (2003) found no significant differences in ICU length of stay or ARDS rates [37]. Similarly, Rixen et al. (2016) observed no significant differences in hospital LOS, transfusion requirements, or complications such as ARDS [39]. In the retrospective cohort study by Yu et al. (2023), hospital LOS was comparable between the groups, as were rates of wound complications [30]. Enninghorst et al. reported no significant differences between groups in transfusion rates, incidence of deep vein thrombosis (DVT), ICU length of stay, hospital length of stay, or mortality. These studies provide the most meaningful insights into the relative safety and efficacy of DCO and EAC, suggesting that when ISS is not a differentiating factor, the outcomes of these techniques are closely aligned.

When looking at the outcomes of the other studies (Table 5) where severity of injury was not accounted for, length of stay (LOS) and complication rates followed expected patterns, with DCO patients consistently experiencing longer ICU and hospital stays and higher rates of complications. Lubken et al. reported an ICU LOS of 8 days in the DCO group versus 3 days in the EAC group (*p* < 0.001) and hospital stays of 26 days compared to 20 days (*p* < 0.001) [21]. Andruszkow et al. found similarly extended ICU LOS for DCO patients, with 27.7 days versus 10.7 days (*p* < 0.001) in their German cohort and 12.2 days versus 7.6 days (*p* = 0.002) in their Australian cohort [32]. Vallier et al. (2010) observed a hospital LOS of 17 days in the DCO group compared to 21 days in the EAC group, though this difference was not statistically significant [19]. Complication rates were also notably higher in DCO groups. Vallier et al. (2010) reported significantly higher pulmonary complications in the DCO group (73 vs. 21, *p* = 0.0024), along with higher infection rates (45 vs. 14, *p* = 0.024) and ARDS rates (34 vs. 8, *p* = 0.019) [19]. Lubken et al. observed higher rates of sepsis (804 vs. 245, *p* < 0.001) and multi-organ failure (2836 vs. 876, *p* < 0.001) in the DCO group [21]. Andruszkow et al. also reported higher rates of infections requiring revision in the DCO group (15 vs. 1, *p* = 0.002) [32]. O’Toole et al. found that transfusion rates were higher in DCO patients (92.9% vs. 58.3%, *p* < 0.05), as were mortality rates (17.9% vs. 2.0%, *p* < 0.05) [28].

The risk of bias assessment, as summarised in Table 1, reveals a spectrum of methodological rigor among the included studies, which may affect the quality and reliability of the synthesised evidence. While several studies demonstrated consistently low risk across all assessed domains, others exhibited significant vulnerabilities, particularly related to the lack of matching and robust selection criteria. These weaknesses raise concerns about potential confounding and selection bias, which could undermine the validity of their findings. Additionally, some studies had uncertain risk due to issues with participant selection, further complicating the interpretation of results

## 4. Discussion

Our findings build upon the foundational concepts of inflammation outlined by Gabay and Kushner, applying these principles to surgical practice, with a particular focus on fracture management in polytrauma patients [47]. Research on trauma physiology has grown significantly, aiming to enhance our understanding and improve guidance for all trauma surgery including fracture care [48]. Nauth et al. highlighted the interplay of immune dysregulation and inflammatory markers in polytrauma [9]. Our study found promising results for the potential use of biomarkers in guiding clinical decision-making frameworks. At the same time, it identifies a significant gap between basic science research and its clinical application, emphasising the need for further exploration to bridge this divide.

Notably, our systematic review is the first to address the challenge of translating academic biomarker research into practical clinical applications. It explores a broader range of biomarkers, including several that remain in the research phase but demonstrate advanced prognostic potential. Evaluating the potential of these emerging biomarkers is crucial to determine whether they warrant scaling for clinical application rather than remaining confined to academic research.

This systematic review identified multiple POC biomarkers with potential for clinical utility in trauma care, focusing on their role in predicting complications such as infection, multiple organ dysfunction syndrome (MODS), and post-operative morbidity. Lactate was particularly well-studied and emerged as a reliable predictor of morbidity and length of stay, with established thresholds enabling clinical application [44,45,46,49]. In addition to POC lactate, several studies demonstrated the ability to use POC tests on immune cells to predict sepsis and infection [11,40,41]. Specifically, monocyte L-selectin, neutrophil L-selectin, neutrophil oxidative burst capacity, and CD16 neutrophils showed significant predictive value in identifying patients at increased risk of these complications [11,40,41].

Our systematic review found many advanced laboratory biomarkers to have significant predictive value for infections, end organ dysfunction, and MODS [24,26,35,36,43,50]. Three studies found IL-6 to significantly predict infection [24,26,43]. Other immunoglobulins found to have significant predictive value were IL-8, IL-7R, IL-17, IL-1RA, IL-10, and IL-23 [24,26,35,36,43]. Besides immunoglobulins, Hmgb1 and leptin were also found to have significant predictive value [24,35]. These biomarkers rely on specialised techniques, such as ELISA and PCR, and they require skilled lab personnel and longer processing times, but they provide detailed insights into the inflammatory and immune responses in trauma patients. Unlike standard markers like lactate, these advanced biomarkers capture the complexity of systemic inflammation, offering significant added value. Their potential to enhance trauma care underscores the importance of further research aimed at improving accessibility and integrating them into clinical practice.

Despite advancements in biomarker research, the integration of these tools into surgical timing decisions remains underdeveloped. Surgical timing in polytrauma patients is a complex interplay of physiological stability and operative risk. Holcomb et al. emphasised the importance of timely intervention in trauma care, noting that delayed surgery in the presence of elevated inflammatory markers may predispose patients to complications like MODS or prolonged ICU stays [51]. Lactate is currently the only biomarker with established thresholds that could potentially guide timing, as supported by studies in Vincent et al.’s systematic review [49]. However, its predictive value in relation to optimal surgical windows has not been fully elucidated.

Timing decisions in trauma surgery are very complex and account for multiple organ systems, including cardiovascular, pulmonary, and renal stability. Although it would be ideal to have a definitive biomarker that provides a clear-cut indication for when to proceed with surgery, such a solution does not yet exist. Entire textbooks such as that by Bassett and Smith highlight the delicate balance required in managing anaesthesia and the decision to determine whether a patient is medically optimised [52]. They specifically address how to properly evaluate a polytrauma patient’s fluid requirements, highlighting the critical role of maintaining hemodynamic stability in surgical planning. Similarly, Chong et al.’s meta-analysis further emphasises the importance of optimising patient haemodynamics prior to surgery [53]. They discuss how goal-directed fluid therapy has improved perioperative outcomes and suggest that lactate kinetics serve as a surrogate for evaluating the physiological readiness for surgery.

In addition to fluid management, the hematologic and cardiovascular systems must also be carefully evaluated, particularly in addressing traumatic coagulopathies, which are a frequent and life-threatening complication in polytrauma patients. As described by Brohi et al., these coagulopathies result from a complex interplay of trauma-induced shock, inflammation, and impaired clotting mechanisms [54]. Their work emphasises the importance of timely administration of blood products and targeted resuscitation protocols to minimise surgical risks. Tobin et al. further stress the role of timely and proper blood product administration in polytrauma patients [55].

Ultimately, a multivariate approach that integrates biomarkers, clinical scoring systems, and real-time physiological monitoring offers the greatest potential for improving trauma care. This approach aligns with the broader framework of comprehensive trauma management outlined in Schwartz’s Principles of Surgery, emphasising the importance of integrating diverse data sources to guide surgical decision making [56]. Our systematic review identified 10 immune system biomarkers, including three POC and seven delayed markers, that predict important perioperative outcomes and provide valuable insights into the inflammatory cascade associated with trauma. These biomarkers hold significant promise for enhancing the understanding of systemic responses and optimising preoperative decision making. Research should focus on prospective trials that evaluate the predictive power of these markers in relation to specific surgical interventions, with an emphasis on reducing complications and optimising recovery. Furthermore, leveraging technological advancements, such as point-of-care testing and artificial intelligence, could accelerate the transition of biomarker research from bench to bedside.

The secondary objective of this review was to compare DCO and EAC and their impact on patient outcomes. Predictably, DCO was consistently associated with higher Injury Severity Scores (ISSs); longer ICU and hospital stays; and increased complication rates such as sepsis, MODS, and ARDS. This aligns with its established role in managing severely injured, unstable patients. Importantly, studies that controlled for ISS, such as Pape et al. (2003), Rixen et al. (2016), and Yu et al. (2023), reported comparable outcomes between DCO and EAC, including ICU length of stay, transfusion requirements, and complication rates. These findings highlight the subjective decision of when to pursue DCO. Ultimately, the decision is often surgeon preference and institution based.

Given this subjectivity and the variability in outcomes, national organisation guidelines and institution specific protocols play a pivotal role in standardising decision making, ensuring that both patient needs and local resources are appropriately accounted for. For example, the 2015 guidelines released by the American College of Surgeons (ACS) and Orthopaedic Trauma Association (OTA) outline specific indications for DCO, such as severe traumatic brain injury and inadequate resuscitation [57]. These guidelines also highlight the appropriateness of DCO in resource-limited settings. While they provide clear recommendations in certain scenarios, much is left open to interpretation by individual institutions. The guidelines emphasise that, although patient physiology and injury severity are key determinants, institutional factors play a critical role in shaping how these protocols are implemented.

Institutions must tailor their protocols to align with their specific resources and capabilities. This involves considering the expertise and comfort level of orthopaedic surgeons in handling complex trauma, the hospital’s capacity for advanced resuscitation measures, the proficiency of anaesthesia teams in managing critically ill patients, and the availability of necessary multidisciplinary teams. For example, a Level I trauma centre with robust resources may adopt protocols emphasising aggressive resuscitation and early definitive fixation, while a smaller facility with limited resources might prioritise stabilisation and timely transfer.

Additionally, variability in patient populations served by institutions can dictate protocol design. Centres treating predominantly older populations with significant comorbidities may need to integrate additional considerations, such as optimising perioperative management and incorporating geriatric expertise into decision-making algorithms. By acknowledging these nuances, institutions can develop biomarker-guided and algorithmic protocols that provide consistency while allowing for flexibility in addressing unique clinical scenarios.

These protocols not only guide clinical decision making but also serve as a foundation for informed consent discussions, helping surgeons effectively communicate the rationale behind choosing DCO or EAC and the potential risks and benefits associated with each approach. Informed consent is a vital consideration when determining whether to pursue DCO or EAC, as both strategies carry significant risks and benefits that can impact a patient’s quality of life [58]. However, trauma patients often lack the capacity to participate in these discussions due to the urgency of their condition, leaving surgeons to rely on surrogate decision-makers or act in the patient’s best interest based on clinical judgment and established protocols.

These situations highlight the surgeon’s responsibility to balance the ethical principles of autonomy and beneficence. While patients may later express frustration over prolonged hospitalisations, additional surgeries, or delayed recovery due to the staged nature of DCO, these measures are often necessary to manage life-threatening conditions and optimise long-term functionality. To minimise potential misunderstandings and enhance trust, preoperative discussions—when possible—should address the potential implications of both DCO and EAC, including the anticipated length of hospitalisation, surgical risks, and how these strategies align with the patient’s values and goals. Surgeons should be prepared to guide these discussions by integrating their expertise; the latest evidence-based research; and an understanding of the patient’s medical, surgical, and social circumstances.

In the end, surgeons must weigh numerous variables—including physiological stability, injury severity, institutional resources, and patient-specific factors—when choosing between DCO and EAC. These decisions must be informed by a thorough understanding of the current literature to ensure that the chosen strategy is the safest and most beneficial for the patient. Although input from patients or their families is invaluable, they often depend heavily on the surgeon’s expertise and guidance. This underscores the importance of fostering a strong physician–patient relationship and ensuring that ethical considerations, such as informed consent, remain integral to trauma care protocols. By addressing these complexities, surgeons can not only optimise outcomes but also maintain the trust and confidence of their patients and their families.

### Limitations

This review has several limitations. Variability in biomarker protocols and thresholds across studies—shaped by patient factors, laboratory differences, and timing—hinders direct comparisons. Many included studies are retrospective, introducing potential bias from confounders such as Injury Severity Score and pre-existing conditions. Heterogeneity in patient selection, institutional protocols, and clinical decision making further complicates comparisons between DCO and EAC. Additionally, differences in trauma care resources across institutions limit generalisability. Future research should prioritise standardised methodologies and multicentre collaborations to enhance consistency and external validity.

## 5. Conclusions

This systematic review is among the first to tackle the challenge of translating academic biomarker research into practical clinical applications. It highlights a broader range of biomarkers, including several that remain in the research phase but demonstrate promising prognostic potential. Evaluating the scalability of these emerging biomarkers is critical to determining their feasibility for clinical use beyond academic research.

Several point-of-care (POC) biomarkers, including lactate, monocyte L-selectin, neutrophil L-selectin, neutrophil oxidative burst capacity, and CD16bright/CD62Ldim neutrophils, show significant utility in predicting complications such as sepsis, infection, and multiple organ dysfunction syndrome (MODS). Among these, lactate stands out for its established thresholds, enabling actionable clinical decision making for morbidity and length of stay. Advanced laboratory biomarkers, such as IL-6, IL-10, IL-7R, HMGB1, and leptin, provide deeper insights into systemic inflammation and immune dysregulation, although their reliance on specialised techniques limits their immediate applicability in acute settings. These advanced biomarkers underscore the importance of further research to enhance accessibility and integrate their use into trauma care protocols.

Despite these advancements, the integration of biomarkers into surgical timing decisions remains underdeveloped. The complexity of determining optimal surgical timing in polytrauma patients—a dynamic interplay of physiological stability and operative risks—highlights the need for refined thresholds and prospective validation of biomarkers. While lactate remains the only biomarker with established thresholds that could potentially guide surgical timing, its role in identifying optimal surgical windows requires further exploration.

Future research should focus on bridging the gap between biomarker discovery and clinical implementation. This includes refining and validating biomarker thresholds, advancing point-of-care testing technologies, and conducting prospective trials to assess their role in improving surgical outcomes. Such efforts will be instrumental in leveraging biomarkers to optimise trauma care, reduce complications, and enhance recovery for polytrauma patients.

## Figures and Tables

**Table 1:** Appraisal using the Cochrane ROBINS-I Risk of Bias Assessment Tool.

Author	Confouding	Selection of Participants	Deviations from Intended Interventions	Missing Data	Measurement of Outcomes	Selection of the Reported Result	Overall Bias	Notes
Li et al. 2024 [18]	+	+	+	+	+	+	Low-risk	
Vallier et al. 2010 [19]	+	?	+	+	+	+	Mod-risk	Unclear selection criteria.
Vallier et al. 2015 [20]	-	+	+	+	+	+	High-risk	Lack of matching.
Lubken et al. 2023 [21]	-	+	+	+	+	+	High-risk	Lack of matching.
Yamamoto et al. 2019 [22]	+	?	+	+	+	+	Mod-risk	Unclear selection criteria.
Enocson et al. 2023 [23]	-	+	+	+	+	+	High-risk	Lack of matching.
Gaski et al. 2019 [24]	-	-	+	+	+	+	High-risk	Lack of matching due to case-series.
Enninghorst et al. 2010 [25]	+	+	+	+	+	+	Low-risk	
Pape et al. 2001 [26]	+	+	+	+	+	+	Low-risk	
Harvin et al. 2012 [27]	-	-	+	+	+	+	High-risk	Lack of matching.
O’Toole et al. 2009 [28]	-	-	+	+	+	+	High-risk	Lack of matching.
Arnold et al. 2024 [29]	+	+	+	+	+	+	Low-risk	
Yu et al. 2023 [30]	+	?	+	+	+	+	Mod-risk	Unclear intervention criteria.
Glass et al. 2017 [31]	+	+	+	+	+	+	Low-risk	
Andruszkow et al. 2013 [32]	+	?	?	+	+	+	Mod-risk	Unclear selection methodology.
Testa et al. 2019 [33]	+	?	+	+	-	+	High-risk	Unclear intervention criteria. Missing ISS.
Frohlich et al. 2018 [34]	+	+	+	+	+	+	Low-risk	
Haupt et al. 2021 [35]	+	+	+	+	+	+	Low-risk	
Jin et al. 2022 [36]	+	+	+	+	+	+	Low-risk	

Table legend: low-risk (+); uncertain (?); **high-risk (-)**.

**Table 2:** Appraisal of randomised control trials using the Cochrane RoB Version 2 Risk of Bias Assessment Tool.

Author	Randomisation Process	Deviations from Intended Interventions	Missing Outcome Data	Measurement of Outcomes	Selection of the Reported Result	Overall Bias	Notes
Pape et al. 2003 [37]	+	+	+	+	+	Low-risk	
Pape et al. 2007 [38]	+	+	+	+	+	Low-risk	
Rixen et al. 2016 [39]	+	+	+	+	+	Low-risk	

Table legend: low-risk (+).

**Table 3:** Biomarkers evaluated.

Study	Biomarker	POC vs. Delayed	Time to Results	Analysis	*p*-Value	Odds Ratio
Briggs [40]	Monocyte L-selectin	POC	~30 min	Sepsis	0.001	1.5
	Neutrophil L-selectin	POC	~30 min	Sepsis	0.005	1.56
Lumsdaine [41]	Neutrophil oxidative burst capacity (fMLP)	POC	~45 min	Post-op infection	0.024	
Pape [42]	IL-6	Delayed	ELISA 4–6 h	Pre vs. post op	<0.05	
	IL-8	Delayed	ELISA 4–6 h	Pre vs. post op	<0.05	
Gaski [24]	IL-10	Delayed	Bioassay 4–6 h	Nosoicomial infection	0.01	
				Degree of organ dysfunction	<0.05	
	IL-6	Delayed	Bioassay 4–6 h	Nosoicomial infection	0.15	
				Degree of organ dysfunction	<0.05	
	IL-1RA	Delayed	Bioassay 4–6 h	Nosoicomial infection	0.13	
				Degree of organ dysfunction	<0.05	
	MCP-1	Delayed	Bioassay 4–6 h	Nosoicomial infection	0.08	
				Degree of organ dysfunction	<0.05	
	IL-8	Delayed	Bioassay 4–6 h	Nosoicomial infection	0.15	
				Degree of organ dysfunction	<0.05	
	HMGB1	Delayed	Bioassay 4–6 h	Nosoicomial infection	0.03	
				Degree of organ dysfunction	<0.05	
	MIG	Delayed	Bioassay 4–6 h	Nosoicomial infection	0.05	
				Degree of organ dysfunction	<0.05	
	IL-23	Delayed	Bioassay 4–6 h	Nosoicomial infection	0.11	
				Degree of organ dysfunction	<0.05	
Hietbrink [43]	IL-6	Delayed	ELISA 4–6 h	Degree of organ dysfunction (ISS)	0.0001	
	Active FC-gammaRII (CD32)	Delayed	ELISA 4–6 h	Degree of organ dysfunction (ISS)	0.120	
	MAC-1	Delayed	ELISA 4–6 h	Degree of organ dysfunction (ISS)	0.092	
Spijkerman [11]	CD16bright/CD62Ldim Neutrophils	POC	~30 min	Infection	0.002	
Frohlich [34]	CD144+	Delayed	Flow cytometry 2–4 h	ISS	<0.001	
	CD42B+	Delayed	Flow cytometry 2–4 h	ISS	<0.001	
Haupt [35]	Leptin	Delayed	ELISA 4–6 h	Multiple organ failure	0.027	
	IL-17A	Delayed	ELISA 4–6 h	Multiple organ failure	<0.05	
Jin [36]	RNA IL-7R	Delayed	PCR 2–4 h	Multiple organ failure	0.001	
Richards [44]	Admission lactate	POC	~30 min	Pulmonary complications	<0.001	
	Preoperative lactate	POC	~30 min	Pulmonary complications	>0.05	
Oladipo [45]	Lactate	POC	~30 min	Post-operative morbidity	0.015	1.305
	Lactate	POC	~30 min	LOS	<0.001	
Nishida [46]	Lactate	POC	~30 min	Post-op complications	0.04	2.64
	Lactate	POC	~30 min	LOS	0.78	

Table legend: Summary of biomarkers evaluated for clinical outcomes, including testing method (POC vs. delayed), result times, analyses, *p*-values, and odds ratios.

**Table 4:** DCO vs. EAC main findings with ISS.

Study	Study Design	Sample Size	Pathology	Criteria for DCO vs. EAC	ISS	Main Findings
Li et al. 2024 [18]	**CC**	120	Lower extremity	Judgement of the on-scene paramedics and doctors of the emergency department.	DCO: 28.1 EAC: 21.3 *p* < 0.001	Damage control surgery is more often selected to treat patients with more severe lower limb injuries, which leads to lower complication rates.
Vallier et al. 2010 [19]	**CC**	645	Pelivs and acetabular fxs	Surgeon preference, delayed patient presentation to our hospital, operating room availability, severe head injuries, or inadequate resuscitation.	DCO: 24.9 EAC: 26.9 *p* = 0.05	Early fixation of unstable pelvis and acetabular fractures in multiply injured patients reduces morbidity and length of intensive care unit stay, which may decrease treatment costs. Further study to ascertain the effects of associated systemic injuries and the utility of physiologic and laboratory parameters.
Vallier et al. 2015 [20]	**PC**	355	Femur, pelvis, acetabular, or spine fractures	Surgeon choice 47 Intensivist choice 6 Medically unstable 5 Operating room unavailable 4 Severe head injury 2 Patient choice 2	DCO: 34 EAC: 25.1 *p* < 0.001	Our EAC protocol recommends definitive fixation within 36 h in resuscitated patients. Early fixation was associated with fewer complications and shorter length of stay. The EAC recommendations are safe and effective for the majority of severely injured patients with mechanically unstable femur, pelvis, acetabular, or spine fractures requiring fixation.
Lubken et al. 2023 [21]	**CC**	12569	Extremity or pelvic fractures	NR	DCO: 30.5 EAC: 25.9 *p* < 0.001	DCO was considerably more often associated with packed red blood cell (pRBC) transfusions (33.9% vs. 13.4%), catecholamine therapy (14.1% vs. 6.8%), lower extremity injuries (72.4% vs. 53.5%), unstable pelvic fractures (41.0% vs. 25.9%), penetrating injuries (2.8% vs. 1.5%), and shock (20.5% vs. 10.8%) and unconsciousness (23.7% vs. 16.3%) on admission.
Yamamoto et al. 2019 [22]	**CC**	19319	Extremity injury	NR	DCO: 9 EAC: 9 *p* < 0.001	DCO was associated with decreased in-hospital mortality in patients with major fractures.
Enocson et al. 2023 [23]	**CC**	419	Pelvic or acetabular fx	NR	NR	Early (within 72 h) definitive surgery of patients with pelvic or acetabular fractures seems safe with regard to risk for reoperation, other adverse events, and mortality.
Enninghorst et al. 2010 [25]	**CC**	45	Pelvic ring fx	Depending on the fracture pattern and the availability of pelvic specialist surgeon, acute temporary external or acute definitive internal fixation is performed.	DCO: 24 EAC: 30 *p* > 0.05	Acute open reduction internal fixation of unstable pelvic ring fractures within 6 h could be safely performed even in severely shocked patients with multiple injuries. The procedure did not lead to increased rates of transfusion, mortality, intensive care unit length of stay, or overall length of stay.
Pape et al. 2003 [37]	**RCT**	35	Femoral shaft	In the emergency room, all patients were randomly assigned to one treatment arm after all injuries had been categorised and the inclusion criteria were met. Patients were randomised to either femoral nailing or damage control by initial external fixation (DCO) and secondary femoral nailing.	DCO: 23.2 EAC: 21.7 *p* > 0.05	A sustained inflammatory response was measured after primary (<24 h) intramedullary femoral instrumentation, but not after initial external fixation or after secondary conversion to an intramedullary implant.
Pape et al. 2007 [38]	**RCT**	165	Femoral shaft	For all patients who met the inclusion criteria, the sealed envelope that contained the type of treatment was opened after completion of the diagnostics and grading of the patient’s status to account for the exclusion criteria.	DCO: 29.0 EAC: 23.3 *p* < 0.001	In stable patients, primary femoral nailing is associated with shorter ventilation time. In borderline patients, it is associated with a higher incidence of lung dysfunctions when compared with those who underwent external fixation and later conversion to intermedullary nail.
O’Toole et al. 2009 [28]	**CC**	227	Femoral shaft	The reason why each patient was selected for DCO cannot be accurately determined.	DCO: 41.4 EAC: 36.6 *p* < 0.05	In the context of resuscitation before reamed intramedullary nailing of femoral shaft fractures, our rate of acute respiratory distress syndrome was lower (*p* < 0.001) than that of a similar study reported in the literature.
Arnold et al. 2024 [29]	**RC**	558	Femur shaft fractures	NR	DCO: 25 EAC: 16 *p* < 0.001	Early definitive fixation (≤24 h) is preferred over delayed definitive fixation (>24 h) for patients with bilateral femur shaft fractures. Although mortality does not differ, overall morbidity and deep venous thrombosis rates, as well as length of hospital and intensive care unit stay, are significantly lower.
Yu et al. 2023 [30]	**RC**	181	Long bone fractures	NR	DCO: 23.0 EAC: 21.9 *p* > 0.05	Delaying fixation may not be necessary to prevent the second hit phenomenon and has not demonstrated any clear benefits.
Rixen et al. 2016 [39]	**RCT**	34	Femoral shaft	All multiple-trauma patients who presented to the participating hospitals with femur shaft fractures were screened. If all inclusion criteria were fulfilled, the patient was randomised and documentation began.	DCO: 39.8 EAC: 38.9 *p* > 0.05	No advantage of the damage control concept could be detected in the treatment of femur fractures in multiple-trauma patients.
Andruszkow et al. 2013 [32]	**RC**	207	Femoral shaft	NR	GermanDCO: 34.4 EAC: 25.5 *p* < 0.001 Australian DCO: 41.0 EAC: 34.0 *p* < 0.001	Despite a higher ISS in the DCO group, there were no differences in posttraumatic complications and survival depending on EAC or DCO treatment.
Testa et al. 2019 [33]	**RC**	147	Femoral shaft	NR	NR	Intramedullay nail is the gold standard for definitive treatment of femoral shaft fractures. In patients with severe associated injuries, external fixation should be a good alternative.

Table legend: Overview of studies comparing damage control orthopaedics (DCO) and early appropriate care (EAC) by study design, sample size, pathology, criteria for treatment selection, Injury Severity Score (ISS), and main findings. CC: case–control study. PC: prospective cohort. RC: retrospective cohort. RCT: randomised control trial.

**Table 5:** Comparison of DCO vs. EAC across cohort studies.

Study	Outcomes	DCO Group	EAC Group	Significance
Li [18]	Infections	2 (5%)	10 (12.5%)	NR
	Delayed union	3 (7.5%)	12 (15%)	NR
	Material failure	2 (5%)	3 (4%)	NR
	Adjusted complication rate	19.50%	30.50%	<0.01
Vallier (2010) [19]	Initial OR time	22	125	*p* < 0.005
	Total OR time	152	125	no sig
	ICU LOS	12	13	no sig
	Hospital LOS	17	21	no sig
	Pna	45	14	0.024
	ARDS	34	8	0.019
	Pulm complication	73	21	0.0024
	Any complication	81	29	0.006
Vallier (2015) [20]	Abdominal injury	31	66	0.0002
	Chest injury	42	167	no sig
	Head injury	43	149	0.08
Lubken [21]	Transfusion	2774	586	<0.001
	Number of surgical procedures	7	4	<0.001
	ICU LOS	8	3	<0.001
	Hospital LOS	26	20	<0.001
	Sepsis	804	245	<0.001
	MOF	2836	876	<0.001
	Inhospital mortality	887	167	<0.001
Yamamoto [22]	In hospital mortality	40	66	0.011
	Mortality 28 days	35	61	0.008
	Pulm complication	17	20	no sig
	Cardiac complication	15	20	no sig
Enocson [23]	Infections	13	14	NR
	Nonunion	3	5	NR
	Nerve injury	37	26	NR
	DVT	9	7	NR
	Intraoperative bleeding	575	720	<0.01
	Hospital LOS	10	10	no sig
Enninghorst [25]	Transfusion	7	5	no sig
	DVT	2	1	no sig
	ICU LOS	4	3	no sig
	Hospital LOS	37	25	no sig
	Mortality	3	0	no sig
Pape (2003) [37]	ICU LOS	3.3	4.8	no sig
	ARDS	0	0	no sig
Pape (2001) [26]	Blood loss intraop	190	210	no sig
	ICU LOS	13.1	12.6	no sig
	Organ dysfunction	9	33	0.01
	ARDS	1	6	0.06
Pape (2007) [38]	ICU LOS (HRs)	298	197	no sig
	Hours of ventilation	209	127	no sig
	ARDS (%)	10	9	no sig
	Sepsis (%)	12	13	no sig
Harvin [27]	Hospital LOS	10	6	<0.001
	Mortality	6	4	0.01
	DVT	10	8	<0.001
	Hospital charges	97,018	59,561	<0.001
O′Toole [28]	Transfusion %	92.9	58.3	<0.05
	ARDS %	0	1.5	no sig
	Mortality	17.9	2.0	<0.05
Arnold [29]	Morbidity	63	36	0.003
	Hospital LOS	15	10	<0.001
	Post procedure LOS	14	11	0.045
	Mortality	6	5	no sig
Yu [30]	Hospital LOS	14.8	15.3	no sig
	Wound complications	5	10	no sig
Glass [31]	Hospital LOS	32	33	no sig
Andruszkow [32]	Infections requiring revision	15	1	0.002
	German-MODS	11.8%	4.6%	no sig
	German-Mortality	17.6	0	0.05
	German-Hospital LOS	54.5	29.8	<0.001
	German-ICU LOS	27.7	10.7	<0.001
	Australian-MODS	12.5	11.7	no sig
	Australian-Mortality	7.5	4.3	no sig
	Australian-Hospital LOS	24.8	20.5	0.03
	Australian-ICU LOS	12.2	7.6	0.002
Rixen [39]	Hospital LOS	32.3	30.2	no sig
	Transfusion requirements	4.7	6.6	no sig
	ICU LOS	21.8	12.4	0.037
Testa [33]	Time to weight bearing	21.8	21.2	no sig

Table 5: Comparison of outcomes between damage control orthopaedics (DCO) and early appropriate care (EAC) across cohort studies. Outcomes include complications, mortality, hospital and ICU length of stay, transfusion requirements, and surgical metrics, with significance levels provided where applicable.

## Supplementary Materials

The following supporting information can be downloaded at: https://www.mdpi.com/article/10.3390/jcm14041329/s1, File S1 and PROSPERO checklist.
