# Supplementary material for: Translating Biomarker Research into Clinical Practice in Orthopaedic Trauma: A Systematic Review
Source: J Clin Med. 2025 Feb 17;14(4):1329. doi: 10.3390/jcm14041329 (PMC11856232; doi:10.3390/jcm14041329)
Supplement: Supplementary file 1 [file jcm-14-01329-s001.zip › jcm-3434159-File S1.pdf]

PubMed Search Strategy:

Search Terms: ((orthopaedic trauma) OR (orthopaedic injuries)) AND ((fracture management) OR (orthopaedic surgical procedures) OR (orthopaedic interventions)) AND ((optimal timing) OR (biomarkers) OR (Damage Control) OR (Early Appropriate Care)) AND ((clinical outcomes) OR (fracture nonunion) OR (complications)) AND (polytrauma) NOT (spine OR imaging)

Filters Applied:

- Language: English
- Study Type: Randomized Controlled Trials, Cohort Studies, Case-Control Studies
- Date Range: All available studies up to December 2024

MEDLINE Search Strategy:

Search Terms: ("orthopaedic trauma" OR "orthopaedic injuries") AND ("fracture management" OR "orthopaedic surgical procedures" OR "orthopaedic interventions") AND ("optimal timing" OR "biomarkers" OR "Damage Control" OR "Early Appropriate Care") AND ("clinical outcomes" OR "fracture nonunion" OR "complications") AND "polytrauma" NOT ("spine" OR "imaging")

Filters Applied:

- Humans only
- English language
- Exclusion of review articles to focus on primary research
- Inclusion of studies with adult patient populations (age ≥18 years)

Google Scholar Search Strategy:

Search Terms:

("orthopaedic trauma" OR "orthopaedic injuries") AND ("fracture management" OR "orthopaedic surgical procedures" OR "orthopaedic interventions") AND ("optimal timing" OR "biomarkers" OR "Damage Control" OR "Early Appropriate Care") AND ("clinical outcomes" OR "fracture nonunion" OR "complications") AND "polytrauma" NOT ("spine" OR "imaging") NOT book

Search Parameters:

- Results were sorted by relevance and publication date
- The first 213 results were screened manually to identify relevant peer-reviewed studies
- Textbooks and instructional articles were excluded
- Studies from predatory or non-indexed journals were excluded

Study Selection Process:

The search results from each database were exported into a reference manager, and duplicates were removed. Two independent reviewers screened titles and abstracts for relevance, followed by full-text assessment based on predefined inclusion and exclusion criteria. Disagreements were resolved by consensus with a senior reviewer.

Rationale for Database Selection:

- **PubMed** was chosen for its extensive coverage of biomedical literature, including clinical trials and observational studies.
- **MEDLINE** provided additional indexed studies with comprehensive metadata.
- **Google Scholar** was included for its broad literature coverage, allowing identification of additional studies not indexed in traditional databases.

This multi-database approach ensured that we captured a broad range of relevant literature, minimizing the risk of publication bias and enhancing the robustness of our systematic review findings.
